# Voluntary intake of non-caloric sweetener drives conditioned bottle-position preference in mice

**DOI:** 10.17912/micropub.biology.001459

**Published:** 2025-01-06

**Authors:** Daisuke H. Tanaka, Tsutomu Tanabe

**Affiliations:** 1 Department of Pharmacology and Neurobiology, Tokyo Medical and Dental University (TMDU)

## Abstract

The present study investigated whether saccharin, a non-caloric sweetener, induces conditioned bottle-position preference in mice. In a two-bottle preference test, the mice initially preferred water from a specific side. When saccharin was introduced on the opposite side, the mice showed increased total intake and a preference for the position of the saccharin bottle. After saccharin removal, the preference for the saccharin-associated position persisted for one day but disappeared by the next day. These findings suggest that saccharin intake drives associative learning between its presence and bottle position, influencing subsequent decision-making and motivation to consume from the previously saccharin-associated position.

**Figure 1. Non-caloric sweetener drives conditioned bottle-position preference:**
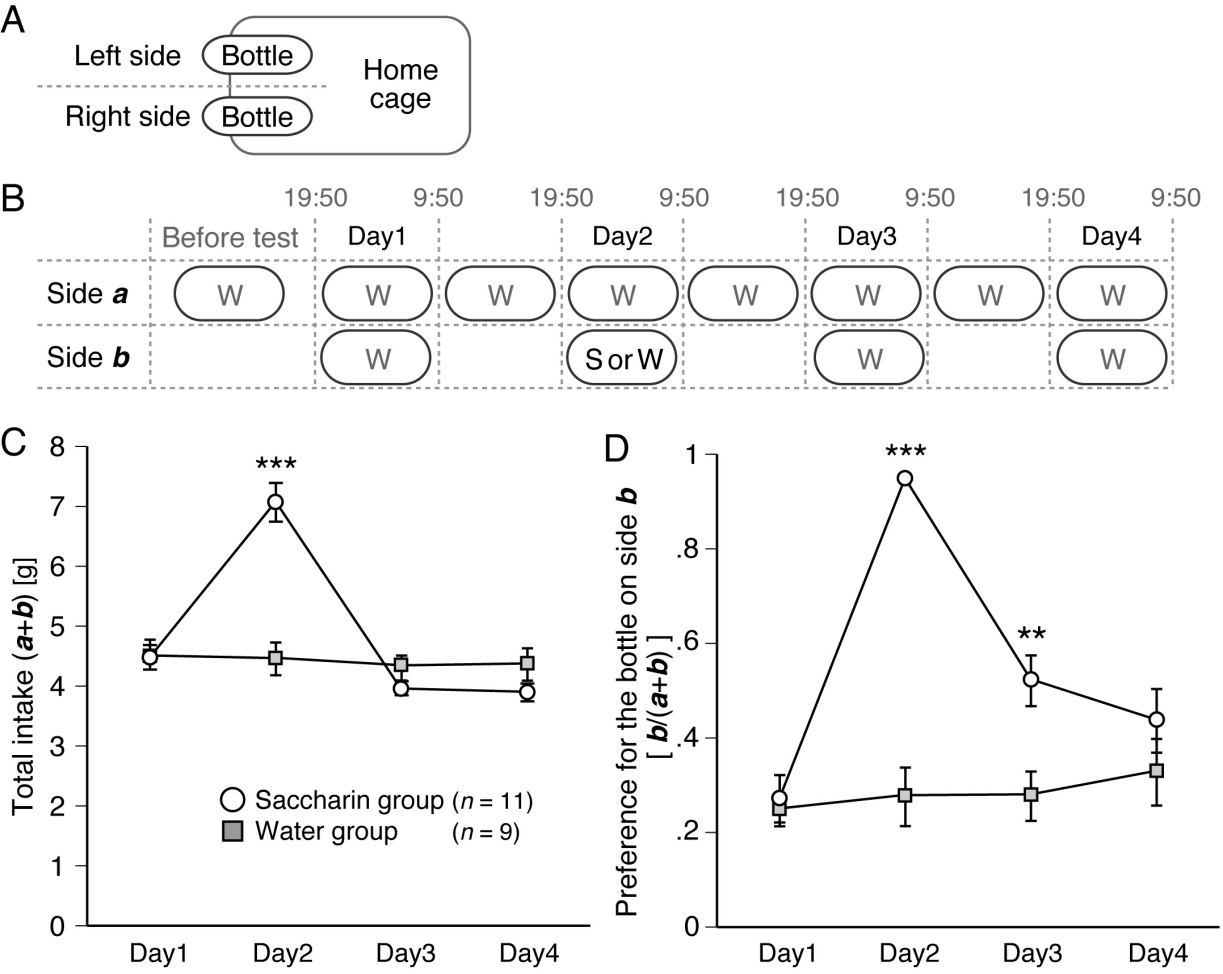
(A) Schematic of the experimental setup. The bottles were placed on the left or right sides of the home cage. (B) Schematic of experimental design and schedule. The side placed by the water bottle for 1-2 wk before the test was assigned to side *a* , and the other left or right side was assigned to side *b* . Two-bottle preference tests were conducted overnight (19:50-9:50 on the next day) on Day1-4. (C) Total intake of water and saccharin solution from two bottles on both sides: *a* and *b* ( *a* + *b* ). Open circles and closed rectangles indicate data from the saccharin ( *n* = 11 mice) and water groups ( *n* = 9 mice), respectively. (D) Preference for the bottle on side *b.* The intake from the bottle on side *b* was divided by the total intake ( *a* + *b* ). Open circles and closed rectangles indicate same meanings as C. W, water; S, saccharin. ** *p* < 0.01, *** *p* < 0.001 (two-tailed Mann-Whitney U test).

## Description

A non-caloric sweetener such as saccharin has been shown to drive conditioned preference for a place (conditioned place preference) (Stefurak and van der Kooy 1992) and a flavor (conditioned flavor preference) (Holman 1975; Fedorchak and Bolles 1987; Grigson et al. 1998) . However, it remains unclear whether the non-caloric sweetener also drives conditioned preference for the position of the bottle containing it (conditioned bottle-position preference). To test this, we performed a two-bottle preference test in which mice had access to two bottles placed on the left and right sides of the home cage ( Fig. 1A ). Before the test, the mice were habituated to a water bottle placed on either the left or right side (which was assigned as side *a* ) ( Fig. 1B ). On Day 1, two water bottles were placed on both sides *a* and *b* , overnight. The mice took ~4.5g of water from the two bottles in total ( Fig. 1C ), and showed a preference for water from the bottle on side *a* ( Fig. 1D ). On Day 2, the content of a bottle on side *b* was changed from water to saccharin solution in some mice (saccharin group) and unchanged in other mice as controls (water group) ( Fig. 1B ). The saccharin group showed significantly higher total intake ( Fig. 1C ), and a higher preference for taking a solution from a bottle on side *b* ( Fig. 1D ) compared to that in the water group. On Day 3, the saccharin solution was removed from the bottle, and both bottles contained water in both groups, as was the case on Day 1. The total intake in the saccharin group was comparable to that in the water group ( Fig. 1C ). Notably, the mice in the saccharin group still showed a significantly higher preference to take the solution from the bottle on side *b* compared to the water group ( Fig. 1D ). This preference disappeared on Day 4 ( Fig. 1D ). These data suggest that voluntary intake of saccharin drives conditioned bottle-position preference: both associative learning between saccharin and the position of the bottle containing it and subsequent decision making and/or motivation to take a solution from the bottle placed on the side where saccharin was available before. In a previous study, a different non-nutritive sweetener, 30 mM sucralose, failed to show bottle-position preference in C57BL/6 mice (de Araujo et al. 2008) . In that study, conditioning was performed in food- and water-deprived conditions for 30 minutes daily for 6 consecutive days. Conditioning in the present study (Day 2) was performed with 5.4 mM saccharin for 14 hours in non-deprived conditions. The conditioning in the present study may be successful because the conditioning time was sufficiently long.

## Methods

***Subjects***

Twenty male C57BL/6J mice, ranging in age from 8 to 11 weeks at the beginning of the experiments, were used. All mice were obtained from Japan SLC Inc. (Shizuoka, Japan), and housed individually in clear plastic cages upon arrival. Mice were maintained at 23 ± 1 ˚C under a 12 h light/dark cycles (lights on at 8:00 am) and given *ad libitum* access to food (Labo MR stock; Nosan Corp., Kanagawa, Japan) and water. All the animal experiments were approved (No. 0150384A, 0160057C2, 0170163C, A2017-194A, and A2018-138C4) by the Institutional Animal Care and Use Committee of Tokyo Medical and Dental University, and performed in accordance with the relevant guidelines and regulations.

***Two-bottle preference test***

For 1-2 weeks in advance of the two-bottle preference tests, the mice were given *ad libitum* access to a water bottle placed on the left or right side of the top of the home cage ( Fig. 1A ). The left and right sides of the water bottle were fixed in each cage. The side placed in the water bottle for–1-2 weeks was assigned to side *a* of the cage, and another empty side was assigned to side *b* ( Fig. 1B ). The left/right side of side *a* was counterbalanced between the subjects. All the two-bottle preference tests were conducted in a home cage. Fluid was available through a ball-bearing nozzle attached to 10-mL glass bottles (BrainScience idea. Co. Ltd., Osaka, Japan), held on top of the cage with a spring. Fluid intake was measured to the nearest 0.1 g by weighing the bottles on an electronic balance. The tests were conducted overnight (19:50-9:50 on the next day) on Day1-4 ( Fig. 1B ). The two bottles contained water on Day 1, 3, and 4. On Day2, a bottle on side *a* contained water, and that on side *b* contained 0.1% (w/v) (5.4 mM) saccharin (code#109185; Sigma-Aldrich) solution (saccharin group) or water as a control (water group). During intervals between the tests (9:50-19:50), the mice were given *ad libitum* access to a water bottle on side *a* ( Fig. 1B ).

***Statistical analysis***

All data are expressed as mean ± SEM. SEM is attributable to the variability across different animals. All data were analyzed using a two-tailed Mann-Whitney U test. Statistical significance was set at p < 0.05.
